# Electrocardiogram pattern of some exotic breeds of trained dogs: A variation study

**DOI:** 10.14202/vetworld.2015.1317-1320

**Published:** 2015-11-22

**Authors:** Joydip Mukherjee, Pradip Kumar Das, Prabal Ranjan Ghosh, Dipak Banerjee, Tripti Sharma, Debananda Basak, Sagar Sanyal

**Affiliations:** 1 Department of Veterinary Physiology, Faculty of Veterinary and Animal Sciences, West Bengal University of Animal and Fishery Sciences, Kolkata, West Bengal, India; 2 Veterinary Surgeon, Kolkata Police Dog Squad, Lalbazar, Kolkata, West Bengal, India

**Keywords:** dog, electrocardiogram, mean electrical axis

## Abstract

**Aim:**

The present study has been conducted to evaluate the variation in electrocardiogram (ECG) parameters among different trained breeds of dogs (viz. Labrador, German Shepherd, and Golden Retriever) used for security reasons.

**Materials and Methods:**

The ECG was recorded by single channel ECG at a paper speed of 25 mm/s and calibration of 10 mm=1 mV. The recordings were taken from all the standard bipolar limb leads (Lead-I, II, and III) and unipolar augmented limb leads (Lead-aVR, aVL, and aVF).

**Results:**

Heart rate was found to be highest in Labrador and lowest in German Shepherd. P-wave duration was maximum in Golden Retriever breed and lowest in Labrador. Maximum amplitude of P-wave was found in Labrador followed by German Shepherd and Golden Retriever. There was significantly (p<0.05) higher values of PR interval in German Shepherd compared to other breeds. The variation in QRS duration, ST segment duration, T-wave duration, and T-wave amplitude was found to be non-significant among breeds. Inverted T-waves were most common in Golden Retriever and German Shepherd, whereas positive T-waves were found in Labrador. There was significant (p<0.05) variation in mean electrical axis of QRS complex among different breeds and it ranges from +60° to +80°.

**Conclusion:**

The present study provides the reference values for different ECG parameters to monitor the cardiac health status among Labrador, German Shepherd, and Golden Retriever breeds.

## Introduction

The electrocardiogram (ECG) is the voltage time graph of the electrical activities of the heart and widely used as a powerful non-invasive diagnostic tool for monitoring heart rate (HR), cardiac rhythm, conduction integrity, and electrical axis [1]. Thus, it is used to evaluate different cardiac diseases in human and other animal species such as cattle [2], small ruminants [3], horses [4] as well as non-cardiac illness throughout the world.

In dogs, cardiac arrhythmias and intra-cardiac conduction disturbances are commonplace problems, which can be well elucidated using ECG [5]. But in India, the exotic breeds are extensively used to perform sniffing activities by different security agencies and also reared in pet lovers’ den. The acclimatization in Indian climate refers several cardiovascular adjustments, which need to be investigated. Further, the sniffer dogs are very prone to different cardiac diseases including cardiac arrhythmia, hypertrophy, atrial fibrillation, flutter, and the heart block of different degrees, which could routinely be evaluated and monitored through ECG. Earlier studies have reported reference values for ECG parameters for different breeds of dogs such as Beagle [6], Labrador [5], and German Shepherd [7]. However, a comparative study on the variation in different ECG parameters of different dog breeds is scanty. In this outset, the present work is undertaken to evaluate the variation in ECG parameters among different trained breeds of dogs (*viz*. Labrador, German Shepherd, and Golden Retriever) used for security reasons.

Though the above three exotic breeds are used for various sniffing activities in homogenous routine training mode, wide variations in ECG are reported among breeds due to genetic differences and shape of thoracic cavity [5]. In addition, the basic cardiac parameters of such dogs in the specific geo-climatic region are lacking. It is envisaged that the generated data could be used for future assessment.

## Materials and Methods

### Ethical approval

The experiments on animals including all procedures of this study were approved by Institutional Animal Ethics Committee.

### Experimental animals

The study was conducted in 18 clinically healthy dogs of either sexes divided into three different breeds, namely Labrador (n=6), German Shepherd (n=6), and Golden Retriever (n=6), from Kolkata Police Training Institute, Kolkata, West Bengal, India. The age of animals ranged from 1 to 2 years with average body weight between 18 and 20 kg. Prior to the experiment, all the animals are further screened for any cardiac abnormality.

### Recording of ECG

Electrographic recordings were taken by a single channel ECG (Cardiart^R^) at a paper speed of 25 mm/s and calibration of 10 mm=1 mV. The recordings were taken from all the standard bipolar limb leads (Lead-I, II, and III) and unipolar augmented limb leads (Lead-aVR, aVL, and aVF) on thermo sensitive ECG paper (Cardiart^R^) having a total width of 50 cm and a recording width of 40 mm. Small square represents 0.04 s on horizontal axis. The amplitude of waves, i.e., the voltage is represented in the vertical axis where 1 mm height represents 0.1 mV. At the time of recording, all the dogs were restrained manually without any anesthesia and maintained at standing condition.

### Measurement of HR, complexes, intervals, and mean electrical axis (MEA)

Waves of ECG recorded by Lead II are considered as the typical waves as the depolarization vector is directed toward the electrode of Lead II [8]. Amplitude of P, Q, R, S, and T-waves was measured together with PR interval, QRS interval, and QT interval. HR was calculated by successive R-R interval. The equilateral triangle measurement system using QRS complex of Lead I and Lead III was employed to determine the MEA [9,10].

### Statistical analysis

One-way analysis of variance was used to test the significance among breeds by SPSS software version 15.0 (SPSS, Inc., Chicago, IL, USA).

## Results

**Figure-1:**
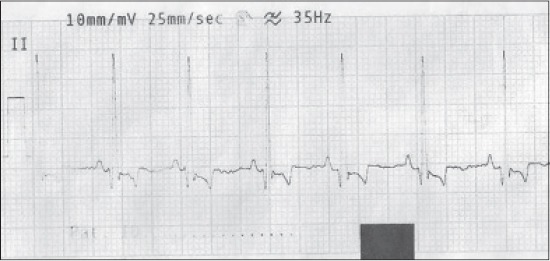
Normal electrocardiograph (Lead-II) of German Shepherd dog.

**Figure-2:**
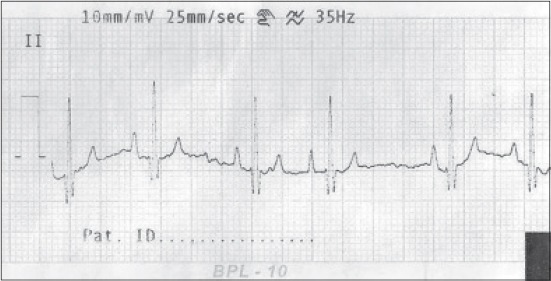
Normal electrocardiograph (Lead-II) of Labrador dog.

**Figure-3:**
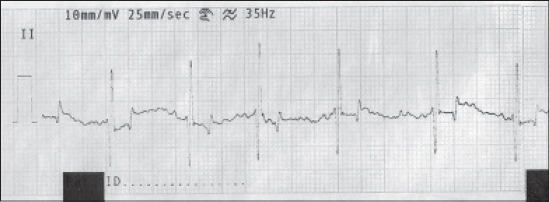
Normal electrocardiograph (Lead-II) of Golden Retriever dog showing inverted T-wave.

Mean ± standard error of different ECG parameters with maximum and minimum values (Lead II) in different breeds of dogs has been presented in Table-1. The ECG of Lead-II has also been depicted in Figures-1-3. There were no significant variations in HR among different breeds. Though HR was found to be highest in Labrador (110.6±5.1 beats/min) followed by Golden Retriever (105.6±5.2 beats/min) and German Shepherd (104.6±5.6 beats/min), P-duration was maximum in Golden retriever (0.053±0.006 ms) followed by German Shepherd (0.046±0.004 ms) and Labrador (0.042±0.001 ms). Maximum amplitude of P-wave was found in Labrador (0.21±0.01 mV) followed by German Shepherd (0.20±0.02 mV) and Golden retriever (0.17±0.03 mV). The PR interval in German Shepherd increased significantly (p<0.05) when compared to other breeds. The variation in QRS duration, ST-duration, T-duration, and T-wave amplitude was found to be non-significant among breeds. The MEA values in different dog breeds under study have been presented in Table-1. In Labrador, Golden Retriever, and German Shepherd, the MEA values were found to be 75.30±6.80, 60.33±7.84, and 82.00±19.43, respectively.

**Table-1:** Mean±SE of different ECG parameters with maximum and minimum values (Lead II) in different breed of dogs.

Breed	ECG parameters (Lead II)									

	HR	P_dur_ (ms)	P_amp_ (mV)	PR_dur_ (ms)	QRS_dur_ (ms)	QT_dur_ (ms)	R_amp_ (mV)	T_dur_ (ms)	T_amp_ (mV)	Mean electrical axis (°)
Labrador	110.6±5.1	0.042±0.0011	0.21±0.01	0.107^a^±0.005	0.04±0.00	0.2±0.007	0.95^a^±0.12	0.04±0.004	0.22±0.016	75.30^a^±6.80
	(88-136)	(0.04-0.06)	(0.1-0.3)	(0.08-0.12)	(0.04-0.04)	(0.08-0.16)	(0.88-1.20)	(0.04-0.08)	(0.2-0.3)	(10-78)
Golden	105.0±5.2	0.053±0.006	0.17±0.03	0.107^a^±0.01	0.04±0.00	0.21±0.006	1.63^b^±0.14	0.04±0.006	0.23±0.033	60.33^b^±7.84
Retriever	(75-125)	(0.04-0.06)	(0.1-0.2)	(0.08-0.12)	(0.04-0.04)	(0.10-0.12)	(1.00-2.00)	(0.04-0.06)	(0.2-0.3)	(52-76)
German	104.6±5.6	0.046±0.004	0.20±0.02	0.127^b^±0.007	0.04±0.003	0.18±0.008	1.14^a^±0.17	0.04±0.008	0.26±0.084	82.00^c^±19.43
Shepherd	(88-125)	(0.04-0.06)	(0.1-0.3)	(0.12-0.16)	(0.04-0.06)	(0.17-0.21)	(1.00-2.00)	(0.04-0.06)	(0.18-0.6)	(56-120)

Values are expressed as mean±SE. Values with different superscript within a column differs significantly (p<0.05). SE=Standard error, ECG=Electrocardiogram, HR=Heart rate

## Discussion

Reference values for ECG parameters for different breeds of dogs in the present study were found to be in accordance with the earlier reports in Beagle dogs [6], Labrador [5], and German Shepherd [7]. In the present study, HR was found to be similar with the reference values as reported by Becker [8]. However, HR is highly variable in dogs and may be altered due to stress and excitation during recording [11]. In the present study, positive P-waves in all the dogs were recorded in Lead-I, II, III, and aVF and negative P-wave were seen in lead aVR and aVL. The amplitude of P-wave representing magnitude of atrial depolarization and spreads from Sinoatrial node to atrioventricular node was slightly higher in Labrador compared to normal as reported earlier [12], but in accordance with other workers [5,13]. Higher amplitude of P-wave in Labrador breeds compared to other two may be explained by the higher HR (110 beats/min) among the three breeds under study. Variation in P-wave amplitude may be explained due to stress exhibited during ECG recording [12,14]. In the present study, Q-wave of QRS complex produced due to ventricular depolarization and the electrical transmission through interventricular septum after P-wave was negatively directed in Lead I, II, III, and aVF, whereas aVR and aVL were positively directed as supported by Avizeh *et al*. [12], but contradictory to Burman *et al*. [13] and Gugjoo *et al*. [5] where they found absence of Q-wave in aVR and aVL lead. Amplitude of R-wave is most commonly used to evaluate the left ventricular function and considered a good indicator for ventricular contractibility [5]. In the present investigation, the amplitude of R-wave was significantly (p<0.05) lower in Labrador breed compared to other breeds in contrary to the reported higher values of R-wave amplitude in Labrador breed by Gugjoo *et al*. [5]. The lower amplitude of R-wave in all the dogs under our study could be explained for their adaptive cardiovascular adjustment during athletic training.

In our present study, the QT interval, which is a dynamic physiological variable that can be affected by the velocities of both the ventricular conduction and repolarization, was in accordance with the earlier reports [5,15-18].

In the present investigation, T-wave amplitude and duration, which is directly related to the repolarization of the ventricular myocardial cells, were found to be within normal range as reported by earlier observation [5,19,20]. In our study, inverted T-wave was very common in Golden Retriever breed (66%) followed by German Shepherd (50%) and less frequent in Labrador (10%). The genesis of T-wave is very complex and the determinants of T-wave polarity were yet to be fully understood [21,22], whereas the altered polarity of T-wave in Lead II may be caused due to elevation of the diaphragm during respiration [23,24].

MEA values of heart in different breed of dogs under study were found to be within normal range as reported earlier [5], whereas significant (p<0.05) variation in MEA was found among breeds where MEA in Golden Retriever was more inclined to vertical axis on the frontal plane, which could be explained due to narrower thorax of Golden Retriever in comparison to Labrador and German Shepherd [11].

## Conclusion

The present study provides the reference values for different ECG parameters in some trained exotic breeds of dogs acclimatized in Indian climate with cardiovascular adjustments. The observation of the present work can be used for monitoring the cardiac health status among Labrador, German Shepherd, and Golden Retriever breeds used for security reasons.

## Authors’ Contributions

SS planned the study. JM, PRG, DB, DB and TS recorded and provided help in analyzing the data. PKD analyzed the data. JM drafted and revised the manuscript under the guidance of PKD and SS. All authors read and approved the final manuscript.
